# Polyphenols alleviate metabolic disorders: the role of ubiquitin-proteasome system

**DOI:** 10.3389/fnut.2024.1445080

**Published:** 2024-08-12

**Authors:** Wei Gu, Guohuo Wu, Guijie Chen, Xianghui Meng, Zhongwen Xie, Shanbao Cai

**Affiliations:** ^1^State Key Laboratory of Tea Plant Biology and Utilization, School of Tea and Food Sciences and Technology, Anhui Agricultural University, Hefei, Anhui, China; ^2^Joint Research Center for Food Nutrition and Health of IHM, Anhui Agricultural University, Hefei, Anhui, China; ^3^The First Affiliated Hospital of University of Science and Technology of China, Hefei, Anhui, China

**Keywords:** polyphenols, metabolic disorders, ubiquitin-proteasome system, nonalcoholic fatty liver disease, lipid metabolism, inflammation

## Abstract

Metabolic disorders include obesity, nonalcoholic fatty liver disease, insulin resistance and type 2 diabetes. It has become a major health issue around the world. Ubiquitin-proteasome system (UPS) is essential for nearly all cellular processes, functions as a primary pathway for intracellular protein degradation. Recent researches indicated that dysfunctions in the UPS may result in the accumulation of toxic proteins, lipotoxicity, oxidative stress, inflammation, and insulin resistance, all of which contribute to the development and progression of metabolic disorders. An increasing body of evidence indicates that specific dietary polyphenols ameliorate metabolic disorders by preventing lipid synthesis and transport, excessive inflammation, hyperglycemia and insulin resistance, and oxidative stress, through regulation of the UPS. This review summarized the latest research progress of natural polyphenols improving metabolic disorders by regulating lipid accumulation, inflammation, oxidative stress, and insulin resistance through the UPS. In addition, the possible mechanisms of UPS-mediated prevention of metabolic disorders are comprehensively proposed. We aim to provide new angle to the development and utilization of polyphenols in improving metabolic disorders.

## Graphical abstract

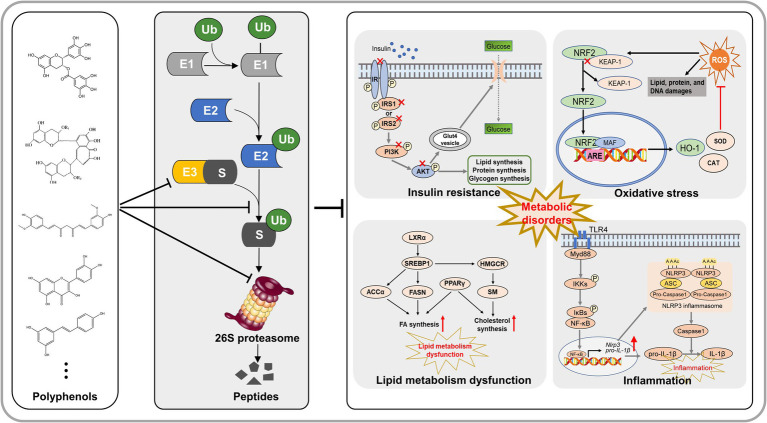

Mechanisms of polyphenols target UPS to prevent metabolic disorders. Polyphenols alleviate metabolic disorders by modulating E3 ubiquitin ligases and the 26S proteasome to mitigate insulin resistance, oxidative stress, abnormal lipid metabolism, and inflammatory responses through regulating the ubiquitin-proteasome system.

## 1 Introduction

Metabolic disorders are a set of syndrome, which include obesity, nonalcoholic fatty liver disease, insulin resistance, and type 2 diabetes. During recent years, metabolic disorders have been recognized as a heavy public health burden (1). Studies have indicated that the morbidity rates are associated with metabolic disorders in the general population, while reaching as high as 75% among individuals with obesity or type 2 diabetes mellitus (T2DM) (2, 3). Nonalcoholic fatty liver disease (NAFLD) is a typical syndrome of metabolic disorders. The constellation of NAFLD symptoms encompasses a spectrum of liver pathology, spanning from beginning hepatic steatosis to more advanced stages including steatohepatitis (NASH), fibrosis, cirrhosis, and ultimately hepatocellular carcinoma (HCC) development (4). The “Two-Hit Hypothesis” was initially proposed as a mechanistic framework to elucidate the initiation and progression of NAFLD (5, 6). Recent researches posits that various factors, including oxidative stress, insulin resistance, lipotoxicity, adipokine secretion by adipocytes, gut microbiota-derived endotoxins, and endoplasmic reticulum (ER) stress, collectively contribute to the progressive development of metabolic disorders (3, 7). Dyslipidemia, inflammation, oxidative stress, and insulin resistance are important factors that contribute to its development (3, 5). Numerous studies have demonstrated that dietary polyphenols can improve dyslipidemia and insulin resistance, possess anti-inflammatory and antioxidant properties, and show significant effectiveness in improving metabolic diseases (8, 9). However, the exact mechanism for the development of metabolic disorders remains incompletely to be understood. Therefore, summarizing the effects of polyphenols on improving metabolic diseases is great significance for exploring the mechanisms of metabolic disorders.

The ubiquitin-proteasome system (UPS) is a crucial cellular pathway accountable for the degradation of undesired or misfolded proteins, and it plays a pivotal function in governing a multitude of cellular processes, encompassing cell differentiation, proliferation, apoptosis, gene expression regulation, DNA repair, and stress response (10, 11). Abnormal UPS activity has been observed in animal models and human patients with metabolic disorders, suggesting its substantial contribution to the development of the diseases (12–14). Dysregulation of the UPS can lead to the buildup of misfolded or damaged proteins, triggering stress pathways, inflammation, and cell death, ultimately contributing to the initiation and progression of metabolic disorders. Therefore, targeting UPS may be a promising therapeutic approach for treating metabolic disorders. Wang et al. (15) demonstrated that Trim16 exerts a beneficial effect in alleviating nonalcoholic steatohepatitis by facilitating the degradation of phospho-TAK1. Yang et al. (16) revealed that RNF5, an E3 ubiquitin ligase, inhibits metabolic disorders progression by targeting HRD1 in the ubiquitin-proteasome pathway. Currently, bortezomib and carfilzomib, two UPS inhibitors, have been approved for clinical use of treating NAFLD (17, 18). Bortezomib has been shown to improve insulin resistance and reduce liver fat accumulation in animal models of NAFLD (19). However, the use of proteasome inhibitors is limited by their potential side effects and systemic toxicity. Therefore, screening nature products targeting UPS emerge as effective and safety therapies for metabolic disorders.

Recently, prevention of metabolic disorders by using dietary regimens and nutraceuticals has attracted much attention. This strategy is driven by the desire to find dietary compounds that can prevent the onset, progression, and severity of metabolic disorders. There is a growing body of evidence that supports the beneficial effects of natural phytochemicals, especially polyphenolic compounds, on suppressing metabolic disorders via protein clearance mechanisms (8, 9). Therefore, this review first summarizes how polyphenols ameliorate metabolic disorders by regulating the UPS, indicating that targeting UPS with polyphenols might have the potential to prevent the pathologies of metabolic disorders. In summarizing the mechanisms by which polyphenols targeting the UPS to ameliorate metabolic disorders, the four possible pathways are proposed: lipid metabolism, inflammation, insulin resistance, and oxidative stress. Hence, the relationship between UPS and these four metabolic pathways is comprehensively summarized. Therefore, this review aims to provide novel theoretical data for the prevention of metabolic disorders through polyphenol targeting UPS.

## 2 Polyphenols protect metabolic disorders by regulating UPS activity

**Figure 1:**
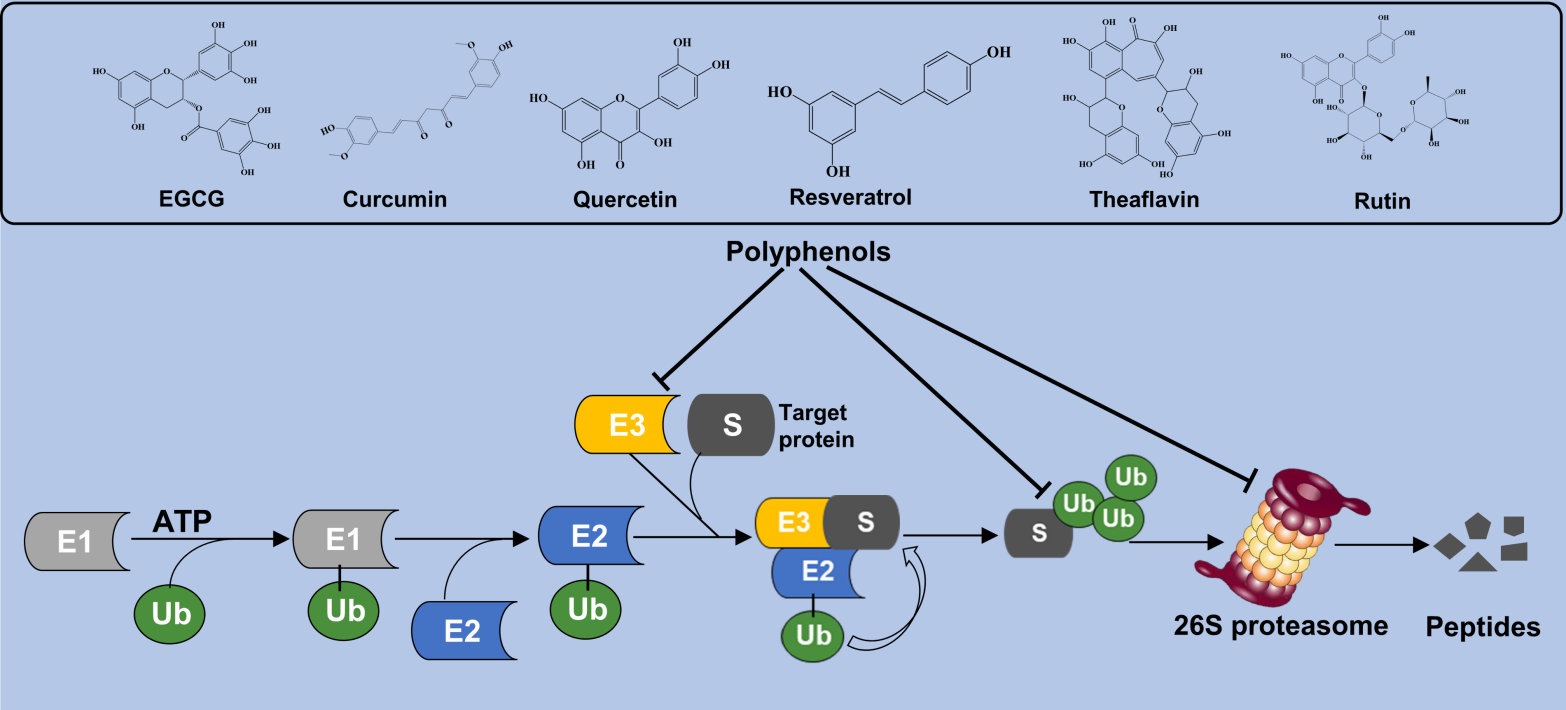
Polyphenols interact the UPS activity. Polyphenolic compounds such as EGCG, curcumin, quercetin, and resveratrol modulate UPS function by either promoting the degradation of proteins indirectly through UPS interactions or directly regulating intracellular proteasome levels.

Polyphenols are a large family of organic compounds found abundantly in natural products. It can be classified into flavonoids, phenolic acids, lignans, and stilbenes based on their phenol ring numbers and structural elements (20, 21). Various phenolic compounds, including EGCG, curcumin, quercetin, resveratrol, theaflavin, and rutin, have been identified for their proteasome-inhibitory activity. Currently, EGCG, quercetin, curcumin, and resveratrol have received the highest research attention, either as standalone agents or in conjunction with other conventional drugs. At present, a substantial body of experimental evidence indicates the remarkable efficacy of these secondary metabolites in combating the progression of NAFLD and metabolic syndrome (22, 23). In addition, the researchers suggested that various polyphenolic compounds targeting different components of UPS can alleviate metabolic disorder. The effect of phenolic compounds on the UPS may be through the following two ways. Firstly, it facilitates the degradation of related proteins indirectly by engaging the UPS. Secondly, it serves as a proteasome regulator, directly controlling the intracellular proteasome levels. In this review, various polyphenols and their influence on UPS activity are summarized and illustrated in Figure 1.

### 2.1 EGCG modulates the UPS process to protect against metabolic disorders

Green tea contains high concentration of polyphenols, and epigallocatechin-3-gallate (EGCG) comprises 50–75% of the total catechin content of green tea (24). Studies have demonstrated that EGCG has potential in the treatment of metabolic disorders such as obesity, insulin resistance, and dyslipidemia, although the underlying mechanisms are not fully understood (25, 26). The recent researches have reported that EGCG can modulate E3 ligase and 26S proteasome to regulate lipid metabolism and exhibit antioxidative and anti-inflammatory effect.

#### 2.1.1 EGCG modulates the UPS process to regulate lipid metabolism

**Figure 2:**
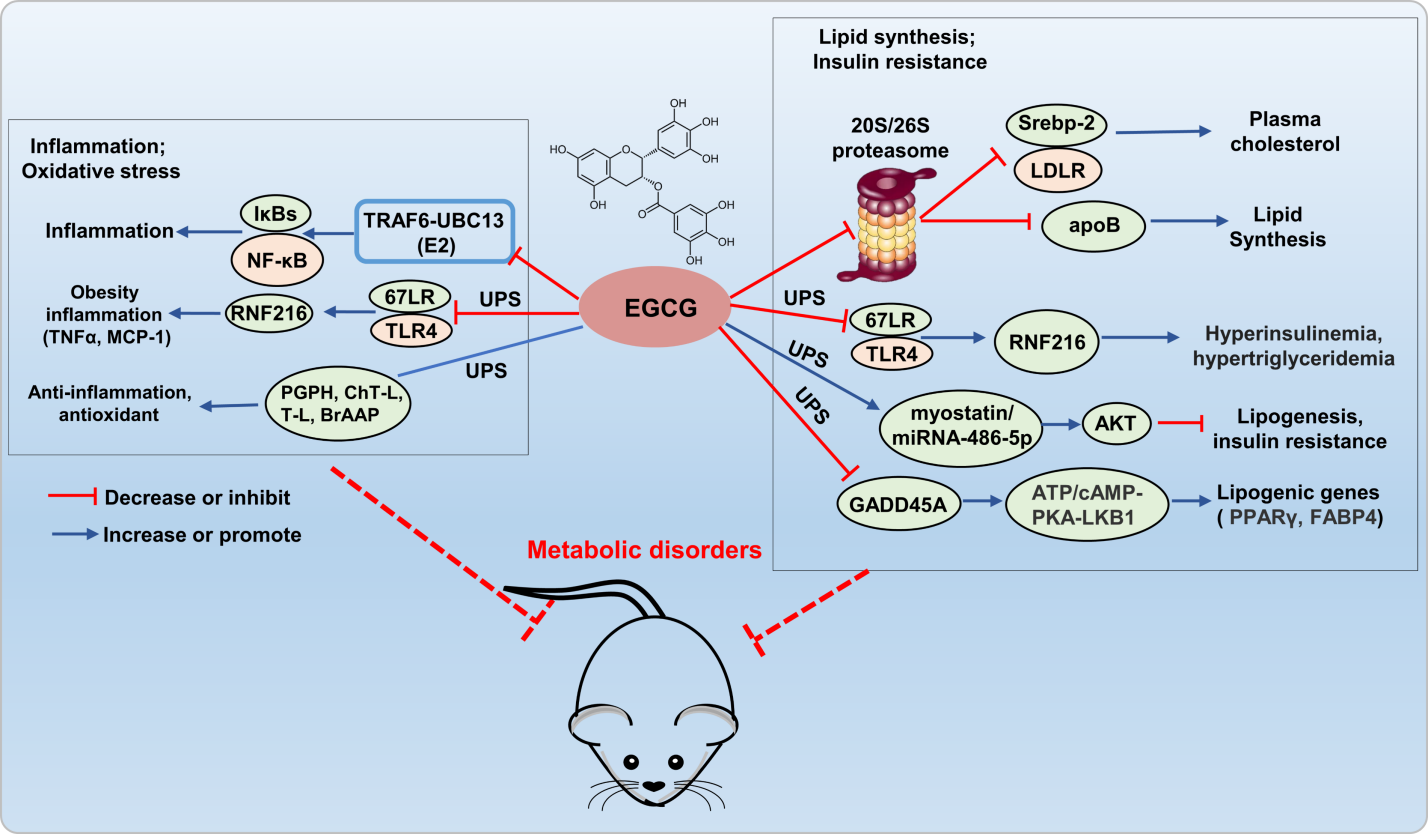
Potential mechanism of EGCG targets UPS to prevent metabolic disorder. EGCG exerts anti-inflammatory and antioxidant effects by regulating E2 (TRAF6-UBC13), E3 (67LR/TLR4), and the 20S proteasome (PGPH, ChT-L, T-L, and BrAAP) within the UPS. It inhibits 20S/26S proteasome, E3 (67LR/TLR4), and GADD45A (interacting with E3 ligase TRIM25), reducing adipogenic gene expression, thus improving lipid metabolism disorders and insulin resistance.

Epigallocatechin-3-gallate has been shown to inhibit the ubiquitin/proteasome-mediated degradation of active SREBP-2, leading to the upregulation of LDLR, which potentially contributed to its fatty liver-improving, cholesterol-lowering, and heart disease-preventive effects (27). A research has reported that EGCG (50 μM) inhibits the secretion of apolipoprotein B-100 (apoB) in HepG2 cells through a proteasome-independent pathway, thereby reducing lipid synthesis (28). Kumazoe et al. demonstrated that EGCG3'”Me served as a natural agonist for the 67-kDa laminin receptor (67LR), effectively attenuated Toll-like receptor 4 (TLR4) expression through the upregulation of E3 ubiquitin-protein ring finger protein 216 (RNF216), thereby mitigating high insulinemia and hypertriglyceridemia induced by high-fat/high-sugar conditions. The study confirmed that EGCG3”Me acts as a natural agonist of 67LR, showing a dose-dependent effect, and this was validated in the HF/HS mouse model. Therefore, 67LR serves as an attractive target, providing strong support for developing EGCG3”Me into a therapeutic agent for hyperinsulinemia and hypertriglyceridemia. EGCG restores AKT activity in skeletal muscle through the myo-statin/miRNAs/ubiquitin-proteasome (miRNA-486-5p) signaling pathway, alleviates age-related muscle mass loss and insulin resistance (29). DNA damage-inducible alpha (GADD45A) is a key regulatory factor in intramuscular fat (IMAT) infiltration, induces ubiquitination and degradation of ATP synthase F1 subunit alpha (ATP5A1) through recruiting the E3 ubiquitin ligase TRIM25, inhibits the cAMP/PKA/LKB1 signaling pathway, upregulates lipogenic gene expression, and promotes adipogenesis in intramuscular preadipocytes. And EGCG effectively reduces lipogenic gene expression by inhibiting GADD45A (30). Hence, EGCG holds promising potential for ameliorating metabolic disorders by addressing lipid metabolism imbalance and insulin resistance through modulation of the UPS system (Figure 2). However, the regulation of lipid disorders and insulin resistance by EGCG through the UPS lacks further in-depth research, limits its development as a drug.

#### 2.1.2 EGCG modulates the UPS process to exert antioxidative and anti-inflammatory effect

Epigallocatechin-3-gallate can also directly interact with E3-TRAF6 ubiquitin ligases, which leading to the attenuation of TRAF6-UBC13 (E2) interaction and inhibition of p65 and p50 translocation, consequently deactivating the NF-κB pathway (31). Kumazoe et al. re-ported that 3″ methlated EGCG inhibited the expression of TLR4 by upregulating RNF216, and further inhibited the expression of tumor necrosis factor α (TNFα) in adipose tissue, and increased serum monocyte chemotactic protein-1 (MCP-1), thus alleviating adipocyte inflammation (32). EGCG exhibited diverse biological activities, including anti-inflammatory, antioxidant, antiviral, and antibacterial effects, achieved through interactions with the PGPH, ChT-L, T-L, and BrAAP subunits of constitutive and immuno-proteasomes, modulated the functionality of the 20S proteasome, thereby conferred antioxidant, anti-inflammatory, and related functionalities (33). Therefore, EGCG exerted antioxidative and anti-inflammatory effects by modulating the UPS system, which demonstrating promising potential as a novel therapeutic agent for the treatment of metabolic disorders (Figure 2).

### 2.2 Curcumin modulates UPS process to alleviate metabolic disorders

Curcumin, a diaryl-heptanoid and a member of curcuminoids, naturally presents in turmeric. Due to its plentiful antioxidant and anti-inflammatory properties, there is considerable interest in exploring the therapeutic potential of curcumin for various metabolic disorders (34). Previous studies have demonstrated that curcumin can improve lipid metabolism abnormalities and insulin resistance, and exhibit significant anti-inflammatory effects by regulating E3 ligase and the 26S proteasome.

#### 2.2.1 Curcumin modulates the UPS process to improve lipid metabolism and insulin resistance

**Figure 3:**
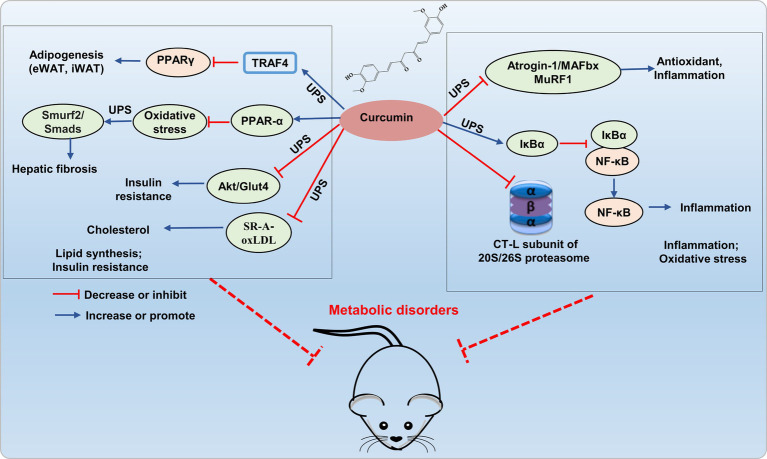
Potential mechanism of curcumin interferes UPS to alleviating metabolic disorder. Curcumin improves lipid accumulation by inhibiting E3 ubiquitin ligases (TRAF4, Smurf2/Smad) and reducing Akt/GLUT4 and SR-A/oxLDL expression via UPS inhibition, thereby enhancing insulin resistance and reducing cholesterol accumulation. It also hinders 26S proteasomal degradation of IκBα, thereby inhibiting NF-κB activation, and inhibits E3 ligases atrogin-1/MAFbx and MuRF1 to decrease inflammatory factor expression.

Recent research indicates that curcumin prevents obesity complication by inhibiting TRAF4-induced ubiquitylation. TRAF4, functioning as an E3 RING ubiquitin ligase, facilitates the degradation of the adipocyte differentiation regulator PPARγ through the ubiquitin-proteasome pathway, thereby impeding adipogenesis (35). Curcumin inhibited the UPS function in 3 T3-L1 adipocytes, activated autophagy, reduced protein kinase B (Akt) protein levels, and concurrently inhibited insulin-stimulated Akt phosphorylation and membrane translocation of glucose transporter type 4 (GLUT4), which leading to the amelioration of insulin resistance (36). In the TGF-β (2 ng/mL) induced BNL CL.2 model, curcumin (20 μM) mitigated hepatic oxidative stress through autophagy activation by inhibiting TGF-β/Smad (Smad2 and Smad3) signaling transmission, inducing ubiquitination and proteasomal degradation of Smad ubiquitin regulatory factor 2 (SMURF2), thereby alleviated hepatic fibrosis by increasing hepatic cell autophagy (37). This discovery confirms that curcumin can mediate hepatocyte autophagy through the UPS pathway to alleviate liver fibrosis, as demonstrated in both animal and cell studies. This provides theoretical support for the development and use of curcumin in liver-protective drugs. In addition, curcumin inhibited scavenger receptor class A (SR-A)-mediated oxidized low-density lipoprotein (oxLDL) uptake through the ubiquitin-proteasome-calpain pathway, and promoted ATP-binding cassette transporter (ABC) A1-dependent cholesterol efflux, thereby retarding cholesterol accumulation in apoE^−^/^−^ mice (38). Therefore, curcumin shows greatly potential in improving metabolic disorders by addressing the imbalance of lipid metabolism and insulin resistance through the modulation of the UPS (Figure 3).

#### 2.2.2 Curcumin regulates the UPS process to exert anti-inflammatory effects

Curcumin exerts inhibitory effects on the activation of the proinflammatory transcription factor NF-κB by hindering the 26S proteasomal degradation of IκBα. Curcumin improved skeletal muscle atrophy in type 1 diabetic mice by inhibiting the muscle-specific ubiquitin E3 ligases atrogin-1/MAFbx and MuRF1, oxidative stress, and inflammatory (NF-κB, TNF-α, and IL-1β) (39). In addition, curcumin is suggested to regulate UPS by inhibiting the proteasome protease activity (40, 41). Curcumin’s carbonyl carbons directly interact with the hydroxyl group of the amino-terminal threonine residue within the proteasomal CT-L subunit found both in 20S proteasome and cellular 26S proteasome (42). Interestingly, curcumin exhibits a dose-dependent effect on the 26S proteasome. In HeLa cells, low doses (1 μM) enhance its activity, while high doses (10 μM) inhibited proteasome activity (43). Research also suggested that curcumin is a powerful inhibitor for COP9 and its associated kinases, such as protein kinase D and casein kinase 2, all of which are closely related to the UPS (44). Additionally, curcumin directly hinder ubiquitin isopeptidases, which are part of the DUB family that responsible for recycling ubiquitin and making it available again for the 26S proteasome system. The regulation of the UPS pathway by curcumin is associated with transcription factors (NF-κB, AP-1, STAT3, HIF-1α, and GADD153/CHOP), which play crucial roles in oxidative stress and inflammation (37, 45). Therefore, curcumin, as an effective inhibitor of proteasome, plays an important role in regulating inflammation and oxidative stress, and has potential value in the prevention and treatment of metabolic disorders (Figure 3). However, most researches on curcumin improving metabolic imbalance through the UPS pathway are based on cell experiments, with fewer studies on animal models.

### 2.3 Quercetin modulates UPS process to alleviate metabolic disorders

Quercetin is a polyphenol distinguished by 3-hydroxyl flavone structure. It exists in several foods, including tea, nuts, apples, berries, onions, shallots, and grapes (46). Extensive researches have consistently demonstrated that quercetin has potent anti-inflammatory and antioxidative properties, and inhibits hepatocyte apoptosis and NAFLD development (22, 47). Current literature has demonstrated that quercetin can improve lipid metabolism and show promising anti-inflammatory effects by modulating the proteasome.

#### 2.3.1 Quercetin modulates the UPS process to improve lipid metabolism

The increased NF-κB activation induced by the ubiquitin-proteasome system may be implicated in the pathogenesis of proteinuria in diabetic nephropathy. Quercetin reduces NF-κB p65 ubiquitination, and significantly lowers fasting insulin, fasting blood glucose, TG, TC, and LDL-C levels, exerts a protective effect against various complications in diabetic rats (48). Liu’s research revealed that quercetin alleviates NAFLD induced by high-fat diet feeding in C57BL/6 J mice by enhancing frataxin-mediated PINK1/Parkin-dependent mitochondrial autophagy (49).

#### 2.3.2 Quercetin regulates the UPS process to exert anti-inflammatory effects

**Figure 4:**
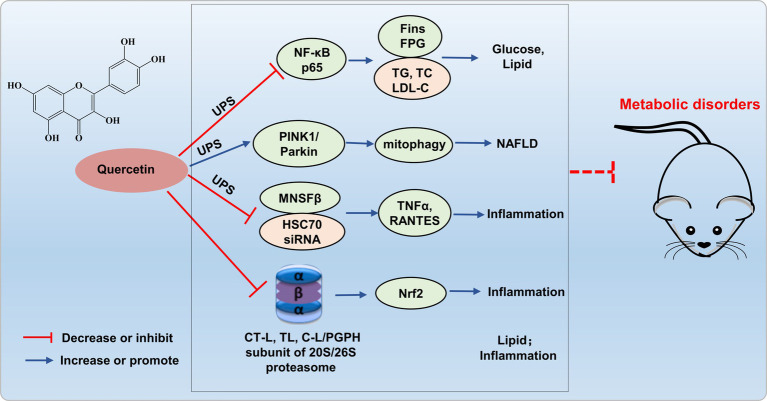
Possible mechanism of quercetin targets UPS to alleviate metabolic disorders. Quercetin reduces NF-κB p65 ubiquitination, enhances frataxin-mediated PINK1/Parkin-dependent mitochondrial autophagy, and inhibits ubiquitin-like protein MNSFβ and HSC70 siRNA, thereby inhibiting inflammation and improving glucose and lipid metabolism disorders.

Quercetin also inhibits the LPS-induced enhancement of TNFα and the production of the C-C chemokine superfamily member RANTES in Raw264.7 cells, mediated by ubiquitin-like protein MNSFβ and HSC70 siRNA (50). Furthermore, quercetin inhibited the activity of all three catalytic subunits of the proteasome, namely CT-L, T-L, and C-L/PGPH, with the CT-L subunit displaying the most potent inhibitory effect (51). Additionally, Sagrario et al. (52) reported that quercetin enhanced proteasome activities and promoted Nrf2 transcription and expression. Quercetin, aside from its modulation of proteasomes to suppress lipid synthesis and anti-inflammatory responses, may constitute an alternative prospective strategy for treating metabolic dis-orders through targeting UPS (Figure 4). Currently, there is limited research on quercetin improving metabolic imbalance through the UPS, and more theoretical studies are needed to enrich the foundational knowledge of quercetin to prevent and treat metabolic disorders.

### 2.4 Resveratrol modulates UPS process to prevent metabolic disorders

Resveratrol, a polyphenol found in grapes and berries, has various biological and pharmacological properties (53). Currently, some studies have reported that resveratrol can improve lipid metabolism imbalances, demonstrates strong anti-inflammatory and antioxidative properties by regulating the UPS, providing protective effects against metabolic disorders.

#### 2.4.1 Resveratrol modulates the UPS process to improve lipid metabolism

**Figure 5:**
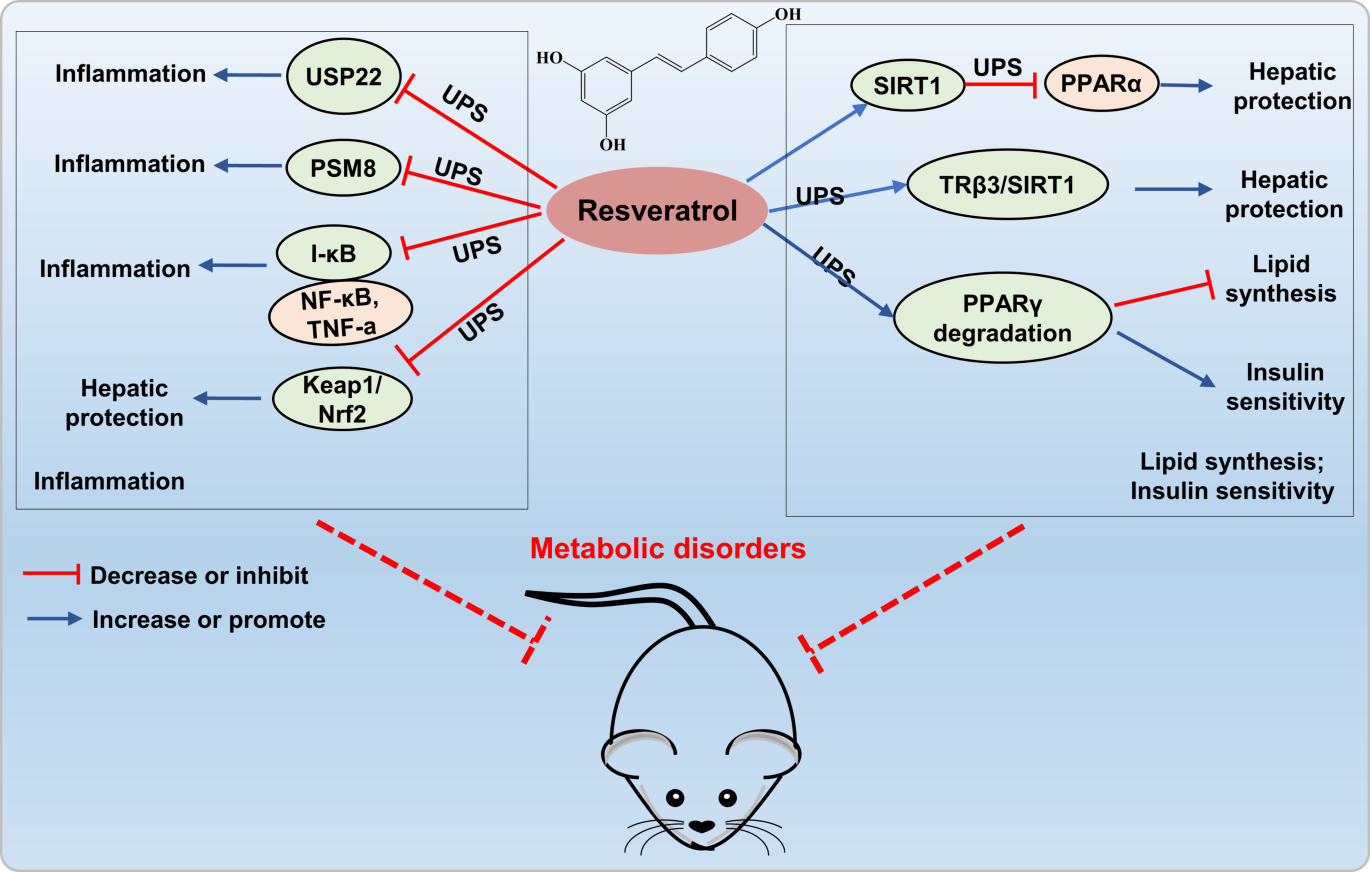
Resveratrol targets UPS to prevent metabolic disorders. Resveratrol inhibits the expression of ubiquitin-specific protease 22 (USP22), proteasome subunit PSMB8, and the Nrf2-Keap1 signaling pathway, thereby suppressing the occurrence of inflammation. Resveratrol activates SIRT1 to decrease PPARα protein levels through ubiquitination, enhances SIRT1 interaction with TRβ1 to facilitate ubiquitin-dependent degradation of TRβ1, and increases ubiquitin-proteasome-dependent degradation of PPARγ protein levels, thereby providing liver protection and improving metabolic disorders.

Resveratrol treated of hepatic cells efficiently activated SIRT1, thereby reducing PPARα protein levels through deacetylation, ubiquitination, and degradation, ultimately improved liver and metabolic dysfunction caused by nutritional deficiencies (54). Resveratrol (100 μM) also exerted a critical role in hepatic metabolic regulation by modulating the triiodothyronine (T3) response through dual target genes TRβ3/SIRT1 in HepG2 cells, facilitating the SIRT1-TRβ1 interaction, promoting TRβ1 deacetylation in the presence of T3, and enhancing ubiquitin-dependent turnover of TRβ1 (55). Floyd’s research revealed that resveratrol modulates PPARγ protein levels in 3 T3-L1 adipocytes by inhibiting PPARγ gene expression and increasing ubiquitin-proteasome-dependent degradation of PPARγ protein, thereby inhibiting lipid synthesis and regulating insulin sensitivity (56). These results suggest that resveratrol may exert a protective effect on the metabolic disorders by modulating lipid metabolism through the UPS (Figure 5).

Researches have shown that resveratrol is a potent proteasome inhibitor and can also function by regulating the UPS (57, 58). Seungkyu et al. (59) reported that resveratrol dose-dependently inhibits HIF-1α and VEGF through the blockade of the PI3K/Akt/mTOR signaling pathway while facilitating proteasomal degradation of HIF-1α. Moreover, He’s research demonstrated a novel synergistic interaction of curcumin and resveratrol (150 mg/kg, 8:2, w/w) when used together to mitigate metabolic-associated fatty liver disease in rats, and the effect is partially attributed to the modulation of the HIF-1 and PI3K/AKT/mTOR signaling pathways (60). Hosseini et al. discovered that resveratrol can mitigate NAFLD by inducing epigenetic modifications of the Nrf2-Keap1 signaling pathway, significantly reducing high glucose-induced triglyceride accumulation in HepG2 cells (61). According to these findings, it is plausible to hypothesize that resveratrol-regulated UPS could be a potential mechanism to improve NAFLD and alleviating metabolic disorders (Figure 5). However, the exact mechanism of resveratrol regulating UPS is still unknown. Further research to unveil how resveratrol regulate UPS to prevent metabolic disorders is needed.

#### 2.4.2 Resveratrol regulates the UPS process to show anti-inflammatory and antioxidative effects

Moreover, resveratrol can also elicit anti-inflammatory and antioxidant effects through the UPS. Shi’s study revealed that resveratrol not only inhibits the overexpression of ubiquitin-specific protease 22 (USP22) and cytotoxicity induced by high D-glucose, but also suppresses the secretion of IL-1β, TNF-α, IL-6, and TGF-β1, providing protection against cell apoptosis and inflammation induced by high glucose (62). Moreover, resveratrol can downregulate the expression of the proteasome subunit PSMB8, thereby inhibiting inflammatory responses (58). The findings indicated that resveratrol exerts anti-inflammatory and antioxidant effects by modulating the UPS, demonstrating its potential value in protection of metabolic disorders (Figure 5).

### 2.5 Other polyphenols modulates UPS process to protect metabolic disorders

Theaflavins are a group of phenolic compounds commonly found in black tea. Studies have demonstrated that theaflavins possess a wide range of health-promoting effects, including antidiabetic, antioxidant, anticancer, and anti-inflammatory activities (63, 64). Theaflavins have been shown to have regulatory effect on UPS. Mizuno et al. (65) reported that TFDG promotes the downregulation of epidermal growth factor receptor (EGFR) through increasing EGFR ubiquitination. And theaflavins may suppress the fatty acid synthase gene through molecular mechanisms that could subsequently lead to the reduction of activity in the EGFR/PI3K/Akt/Sp-1 signal transduction pathways (66). Rutin is a natural phenolic compound present in various plants, including tea, buckwheat, and apples (67). Rutin has been proven to be an important UPS regulator. Research reported that rutin activate the UPS by positively influencing the Nrf2 transcription factor regulators, leading to increased translocation of Nrf2 to the nucleus (52, 68). Rutin additionally hinders carfilzomib-induced oxidative stress and inflammation by acting through the NOS-mediated NF-κB signaling pathway (69). Rutin displayed a multifaceted impact, encompassing the prevention or reversal of metabolic changes such as improved glucose tolerance (70) and reduced abdominal fat pads (71), countering alterations in cardiovascular and hepatic structure (72), as well as mitigating inflammation and oxidative stress in the heart and liver (73–75), while also normalizing liver markers (76). Therefore, utilizing rutin to target UPS could serve as an effective strategy for NAFLD and metabolic disorders treatment.

## 3 UPS modulates critical biological processes affecting metabolic disorders

Polyphenols ameliorate metabolic disorders through the UPS by regulating lipid metabolism, inflammation, oxidative stress, and insulin resistance during disease development. The UPS is responsible for protein degradation and homeostasis, and plays a significant role in the development of metabolic disorders. Consequently, we further review the latest findings by which the UPS affects metabolic disorders by modulating these key pathways, aiming to provide new mechanisms for understanding how polyphenols ameliorating metabolic disorders.

### 3.1 UPS and lipid metabolism

Disruptions of hepatic lipid metabolism are linked to metabolic disorders development (77). Previous researches indicated that UPS crucially regulates lipid metabolism, including cholesterol synthesis, uptake, and efflux (78, 79). The intricate cholesterol synthesis pathway involves over 20 enzymes, with emphasis on pivotal flux-controlling enzymes, such as squalene monooxygenase (SQLE) and 3-hydroxy-3-methylglutaryl-CoA reductase (HMGCR). Researchers have showed that both enzymes undergo ubiquitination and subsequent degradation (80, 81). HMGCR is a key substrate of UPS targeted by statins. And statins can degrade HMGCR and inhibit NASH through ubiquitin-proteasome pathway (82). The E3 ligase in sterol-dependent degradation is gp78 (79, 83), located in the ER membrane. Gp78 first interacts and then promotes the degradation of ER resident proteins, Insig-1 and Insig-2 (84, 85), which is a crucial event for sterol-dependent HMGCR degradation and pre-venting proteolytic activation of sterol regulatory element binding proteins (SREBP) (85). A previous study revealed that Gp78^−/−^ mice exhibit typical characteristics of NAFLD, including lipid degeneration, hepatic inflammation, and fibrosis (86). Moreover, the E3 ligase HRD1 is implicated in the constitutive degradation of HMGCR (87, 88). SQLE converts squalene to squalene-2,3-epoxide, which is a crucial step in cholesterol biosynthesis. Recently, post-transcriptional SQLE regulation gained attention. Researchers found that the E3 ligase that mediating SM ubiquitylation was identified as MARCH6 (89, 90). MARCH6, an ER membrane protein, is part of the ERAD system (90), and loss of MARCH6 activity increases SQLE protein abundance in mammalian cells (89). Moreover, the pathological consequences of P450-ERAD disruption serve as a significant triggering factor for NAFLD (91).

The low density lipoprotein receptor (LDLR) pathway mediates cholesterol imports in mammalian cells (92). E3 ubiquitin ligase-inducible degrader of LDLR (Idol) induce endocytosis and lysosomal degradation of LDLR by polyubiquitinating (93). In mice lacking LDLR, high-fat diet induces degradation of hepatic mitochondrial proteins, leading to oxidative stress, lipid degeneration, and the development of metabolic disorders (94).

Abnormal cholesterol metabolism directly leads to metabolic disorders (95). Cellular cholesterol levels are finely tuned by the interplay of biosynthesis, uptake, and efflux, and are primarily regulated by two transcription factor families, which are liver X receptors α and β (LXRs) (96, 97) and SREBPs (97, 98). LXRs respond to elevated cholesterol levels by activating ABCA1, ABCG1, and the E3 ligase IDOL, subsequently reduce cholesterol burden. LXRs activation by endogenous ligands in response to increased cellular cholesterol induces IDOL, ABCA1, and ABCG1 expression, and reduce cholesterol burden (96, 99). Additionally, ubiquitin-dependent mechanisms control SREBP2 protein stability, processing, and transcriptional activity. In the ER, E3 ligases TRC8, MARCH6, and RNF145 negatively regulate SCAP and SREBP2 (79, 100–102). Furthermore, the mature nuclear SREBP2-N transcription factor, akin to its precursor, is unstable. Phosphorylated SREBP2-N undergoes ubiquitination by the Fbw7-Cullin-1 E3 ligase, leading to proteasomal degradation (103). A previous research indicated that loss of HSP90β promotes the degradation of mSREBPs via Akt-GSK3β-FBW7 pathway, which markedly reduced neutral lipid and cholesterol levels and improved NAFLD and metabolic disorders (104). In summary, ubiquitylation and degradation are crucial processes for maintaining cholesterol homeostasis, impact lipid synthesis (HMGCR, SQLE), uptake (LDLR), efflux (ABCA1, ABCG1), and transcriptional programming (SREBP, LXR).

The UPS also plays a crucial role in regulating fatty acid metabolism. Peroxisome proliferator-activated receptors (PPARγ) are vital transcription factors for regulating lipid and lipid-protein metabolism, undergo ubiquitin-mediated proteasomal degradation facilitated by CHIP (105). Additionally, the E3 ubiquitin ligase NEDD4 stabilizes PPARγ by inhibiting proteasomal degradation (106, 107). USP14, a key proteasome-associated DUB, is crucial for proteome lipid homeostasis (108, 109). Liu et al. (109) highlighted its essential role in hepatosteatosis by stabilizing fatty acid synthase (FASN). Additionally, Hu et al. showed that the E3 ligase TRIM28 mediates FASN ubiquitination and subsequent proteasomal degradation, thus disrupts lipid accumulation in hepatic cells and suppresses NAFLD (110).

**Figure 6:**
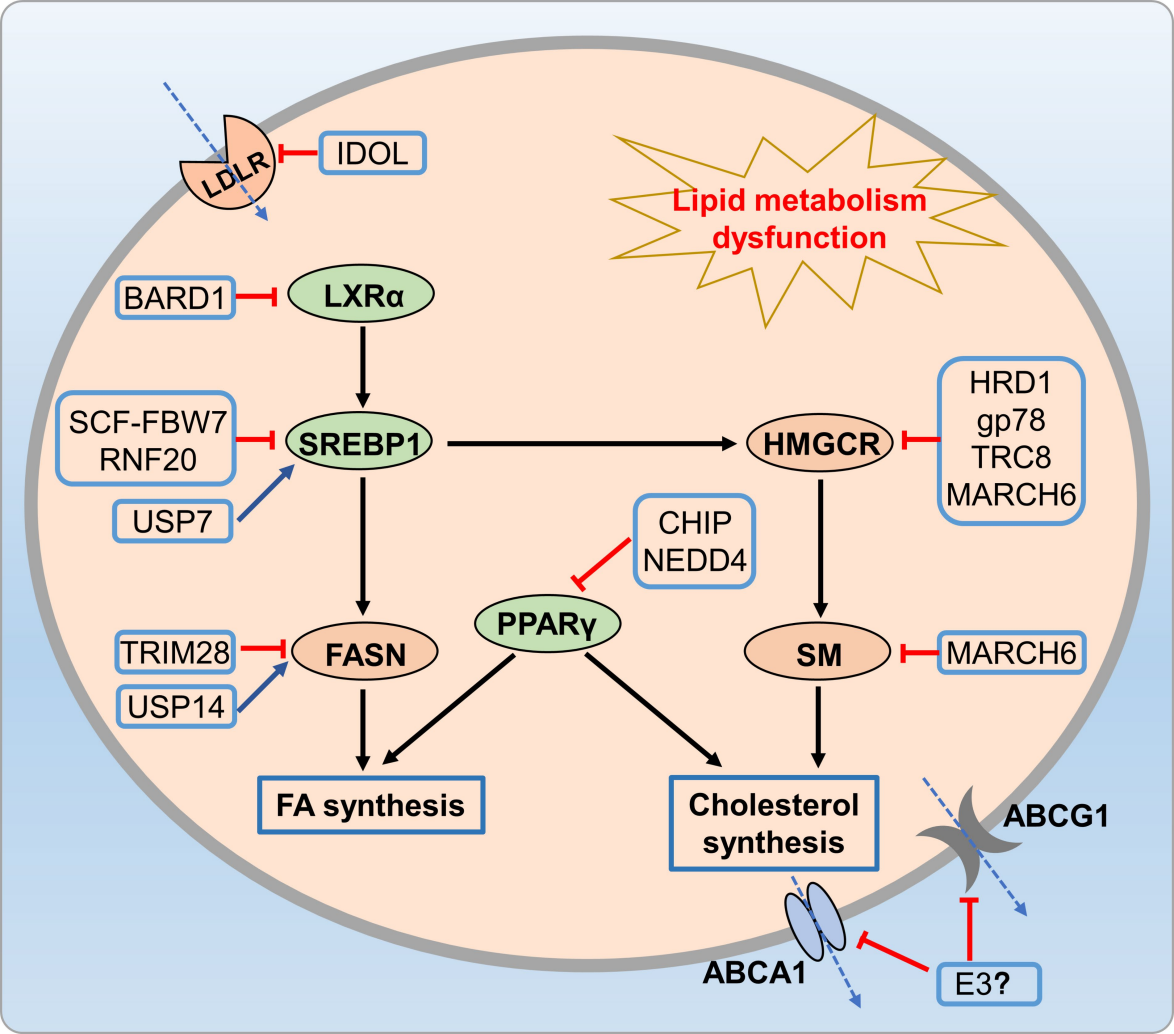
UPS-mediated lipid metabolic disorders. Many E3 ligases (HRD1, Gp78, and MARCH6) reduce cholesterol synthesis by promoting HMGCR ubiquitination and degradation, while CHIP and NEDD4 regulate cholesterol and fatty acid synthesis through ubiquitination of PPARγ. Additionally, E3 ligases like FBW7-Cullin-1, USP14, and TRIM28 reduce fatty acid synthesis by regulating SREBP-1 and FASN.

Therefore, exploring the relationship between the UPS and lipid metabolism has the potential to unveil novel therapeutic approaches for preventing metabolic disorders through dietary polyphenols (Figure 6).

### 3.2 UPS and inflammation

Inflammation is a distinct characteristic of metabolic disorders. The disruption of UPS is associated with inflammation by degrading transcription factors in the inflammatory process (111, 112). Numerous studies emphasize the role of various E3 ubiquitin ligases in regulating inflammation. Cbl-b, Pellino3, and ITCH of E3 Ligases are closely associated with inflammation in metabolic disorders. Cbl-b, a RING E3 ligase, is implicated in protein tyrosine kinase activation, potentially influencing macrophage infiltration and activation (113). Cbl-b over-expression inhibited saturated fatty acid-induced toll-like receptor 4 (TLR4) signaling, as it mediates TLR4 ubiquitination and degradation in macrophages, leading to modulated TLR4 protein levels on the cell surface, thereby improving insulin resistance in the liver (113, 114). The pellino family regulated the TLR/IL-1R signaling pathway, subsequently impacted inflammatory cascades and immune response (115). Pellino3 deficiency in mice exacerbated inflammation, elevated IL-1β expression, and intensified insulin resistance induced by a high-fat diet (116). ITCH, a highly expressed HECT-type E3 ubiquitin ligase, increases anti-inflammatory M2 macrophages, subsequently prevents steatohepatitis when inactive in cells (117). Furthermore, E3 ligase CHIP regulates TLR2/4/7/9 signaling and inhibits NF-κB-mediated inflammation by promoting karyopherin α2 (KPNA2) degradation (118, 119). Conversely, ubiquitin ligases midline1 and siah2 enhance pro-inflammatory cytokine expression, positively regulating NF-κB and MAPK pathways (120, 121).

**Figure 7:**
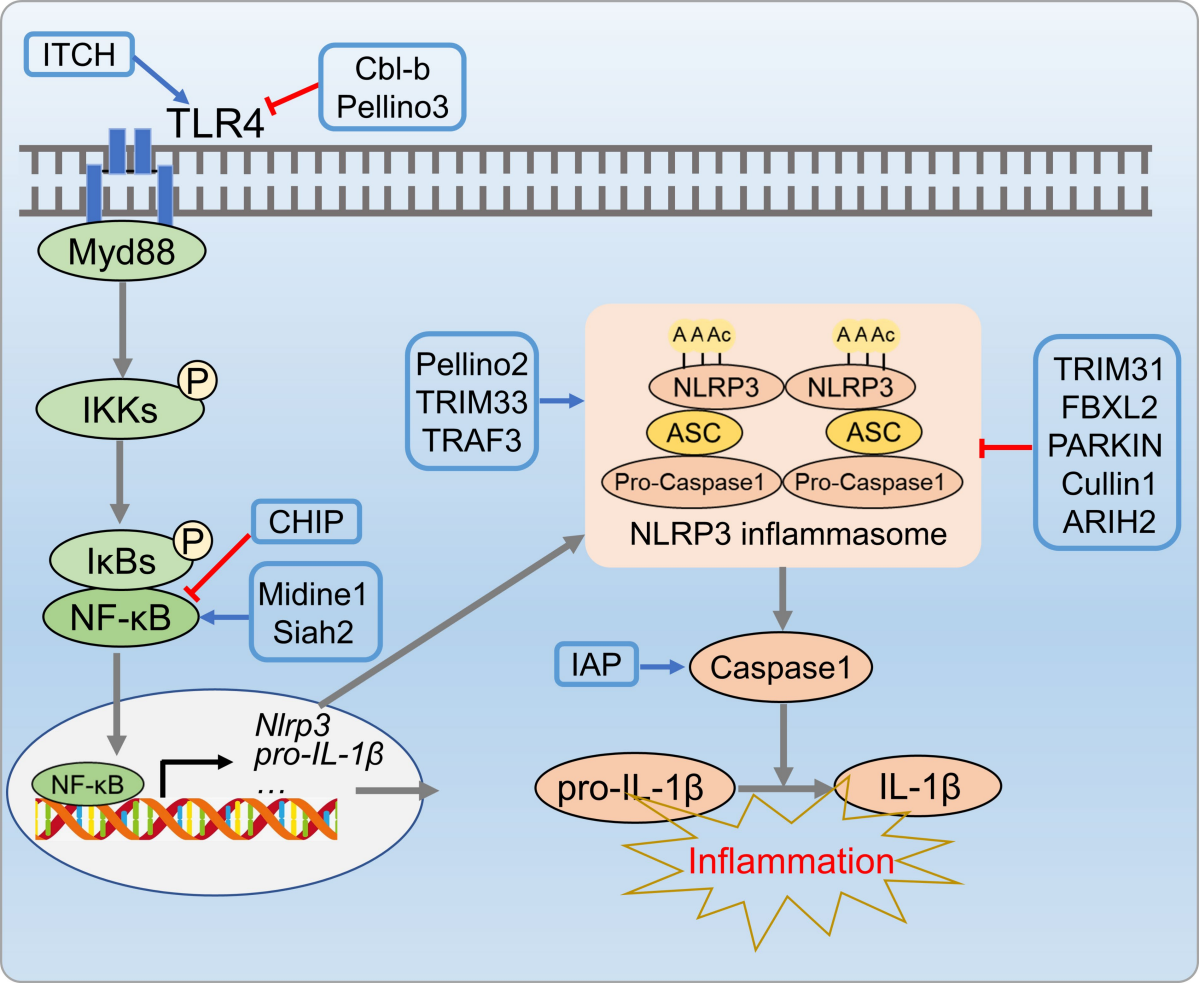
UPS-mediated inflammation processes in metabolic disorders. E3 ligases CL-b, Pellino3, and ITCH E3 inhibit inflammatory cascade reactions by regulating ubiquitination and degradation of TLR4. Additionally, ubiquitin ligases (TRIM31, Parkin, SCF-FBXL2, Cullin, etc.) suppress NLRP3 inflammasome activity by targeting NLRP3, caspase-1, and ASC.

The NLPR3 inflammasome activation has been implicated in NAFLD progression, especially from hepatic steatosis to NASH (122). Thus, precise regulation of inflammasome is crucial to prevent NAFLD progression. The UPS has emerged as a key regulator of inflammasome activation, particularly in the modulating NLRP3 inflammasome complex (NLRP3, ASC, and procaspase-1). Upon activation, procaspase-1 cleaves and releases active caspase-1 that activates IL-1β and IL-18, aggravating hepatic inflammation and contributing to the innate immune response (123). E3 ubiquitin ligases also regulate NLRP3 inflammasome activation by targeting NLRP3, caspase-1, and ASC. TRIM31 binds to NLRP3, induces K48 poly-Ub and promotes NLRP3 proteasomal degradation (124). SCF-FBXL2 ubiquitinates NLRP3 in the resting state, which leading to proteasomal degradation (125, 126). Parkin, an E3 ligase, regulates NLRP3 through deubiquitinase A20, negatively controls priming and activation (127). The newly identified E3 ligase cullin interacts and ubiquitinates NLRP3 during priming, inhibits its activation (128). E3 ligases can also act as positive regulators, as shown by Juliana et al. (129), who demonstrated non-transcriptional priming for NLRP3 activation. Various E3 ligases, including pellino2, TRAF6, and TRIM33, are implicated in non-transcriptional priming of NLRP3. The NLRP3 inflammasome complex is regulated through ASC ubiquitination. NLRP3 and ASC undergo negative regulation through K63 polyubiquitination, resulting in degradation via autophagy, which is a mechanism that controls NLRP3 inflammasomes (130). Additionally, E3 ligases play a role in caspase-1 activation, as shown by Labbe et al. (131), who demonstrated that IAPs induce K63 polyubiquitination of caspase-1, a crucial step for inflammasome activation (Figure 7).

Therefore, targeting the UPS to combat inflammation related metabolic disorders may provide new theoretical support for using dietary polyphenols to ameliorate these conditions.

### 3.3 UPS and oxidative stress

The UPS is crucial for maintaining redox homeostasis. Oxidative stress (OS) is a key contributor to development of metabolic disorders (132). In addition to the main antioxidant defense system, cells use proteolysis and transcription factors to counteract oxidative stress. Nuclear factor erythroid 2-related factor 2 (Nrf2) serves as a pivotal transcription factor activated in response to oxidative stress, directly influencing the onset and progression of NAFLD (133, 134). ROS increases hepatic oxidative stress, and Nrf2 regulates cellular resistance to oxidative damage by controlling antioxidant response element (ARE)-dependent genes (135). Normally, Nrf2 is cytoplasmic, bound to its inhibitor (Kelch-like ECH-associated protein 1Keap1) (136).TheKeap1/Nrf2 complex, involving the Cul3-RbX1 holoenzyme (an E3 ubiquitin ligase from the Cullin-RING box family), leads to Nrf2 ubiquitination and subsequent degradation by the 26S proteasome (136, 137). Oxidative stress causes Keap1 oxidation, which preventing its binding to Nrf2, lead Nrf2 release into the cytosol (137, 138). TRIM25 targets Keap1 directly through ubiquitination and degradation, resulting in Nrf2 activation, thereby enhancing cellular oxidative stress responses and causing damage to liver tissues (139).

**Figure 8:**
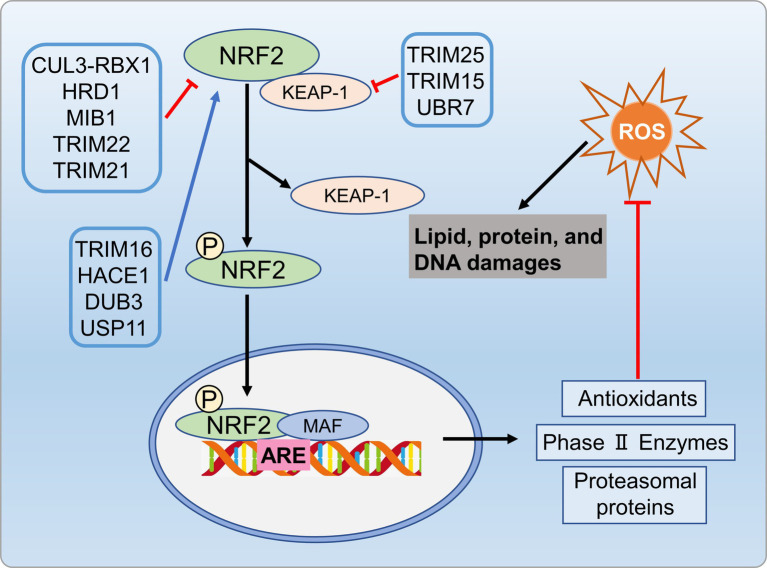
UPS regulates oxidative stress process involved in metabolic disorders. Specific E3 ubiquitin ligases like HRD1, MIB1, and TRIM22 promote NRF2 ubiquitination and degradation, inhibiting antioxidant pathways. Conversely, ubiquitinases TRIM16 and HACE1, as well as deubiquitinases DUB3 and USP11, enhance antioxidant pathways by regulating NRF2 protein stability. Additionally, TRIM25 and TRIM15 ubiquitinate and degrade Keap1, activating NRF2 and enhancing antioxidant defenses.

Various kinases, including MAPKs, protein kinase C (PKC), or PI3K, phosphorylate Nrf2, and promote its nuclear translocation (140). In the nucleus, Nrf2 binds to the ARE in the gene promoters, enhancing transcription for the antioxidant response (138, 141). Specific E3 ubiquitin ligases like HRD1, MIB1, and TRIM22 facilitate NRF2 ubiquitination and degradation, inhibiting the antioxidant pathway (142–144). In cirrhotic livers, the activation of the XBP1-Hrd1 arm of ER stress transcriptionally upregulated Hrd1, leading to increased ubiquitination and degradation of Nrf2, thereby attenuating the Nrf2 signaling pathway (142). Furthermore, E3 ubiquitin ligase TRIM21 negatively regulates the p62-Keap1-Nrf2 antioxidant pathway by ubiquitinating p62. Conversely, TRIM16 and HACE1 enhance NRF2 protein stability by competitively binding, thereby promoting the antioxidant pathway (145, 146). Deubiquitinases DUB3 and USP11 in-crease NRF2 stability and transcriptional activity by reducing NRF2 ubiquitination (147, 148). Additionally, USP enzymes target Keap1 to influence oxidative stress pathways. TRIM25 and TRIM15 directly ubiquitinate and degrade Keap1, activating Nrf2 and enhancing the antioxidant defense system (139, 149). In metabolic disorders condition, impaired proteasome activity, compromised Nrf2 function, and inadequate response to oxidative stress expedite disease progression. Modulating the UPS to enhance antioxidant enzyme expression, to control Nrf2 activation, and to improve proteasome function can be benefit metabolic disorders by alleviating ROS-related effects (Figure 8).

### 3.4 UPS and insulin resistance

**Figure 9:**
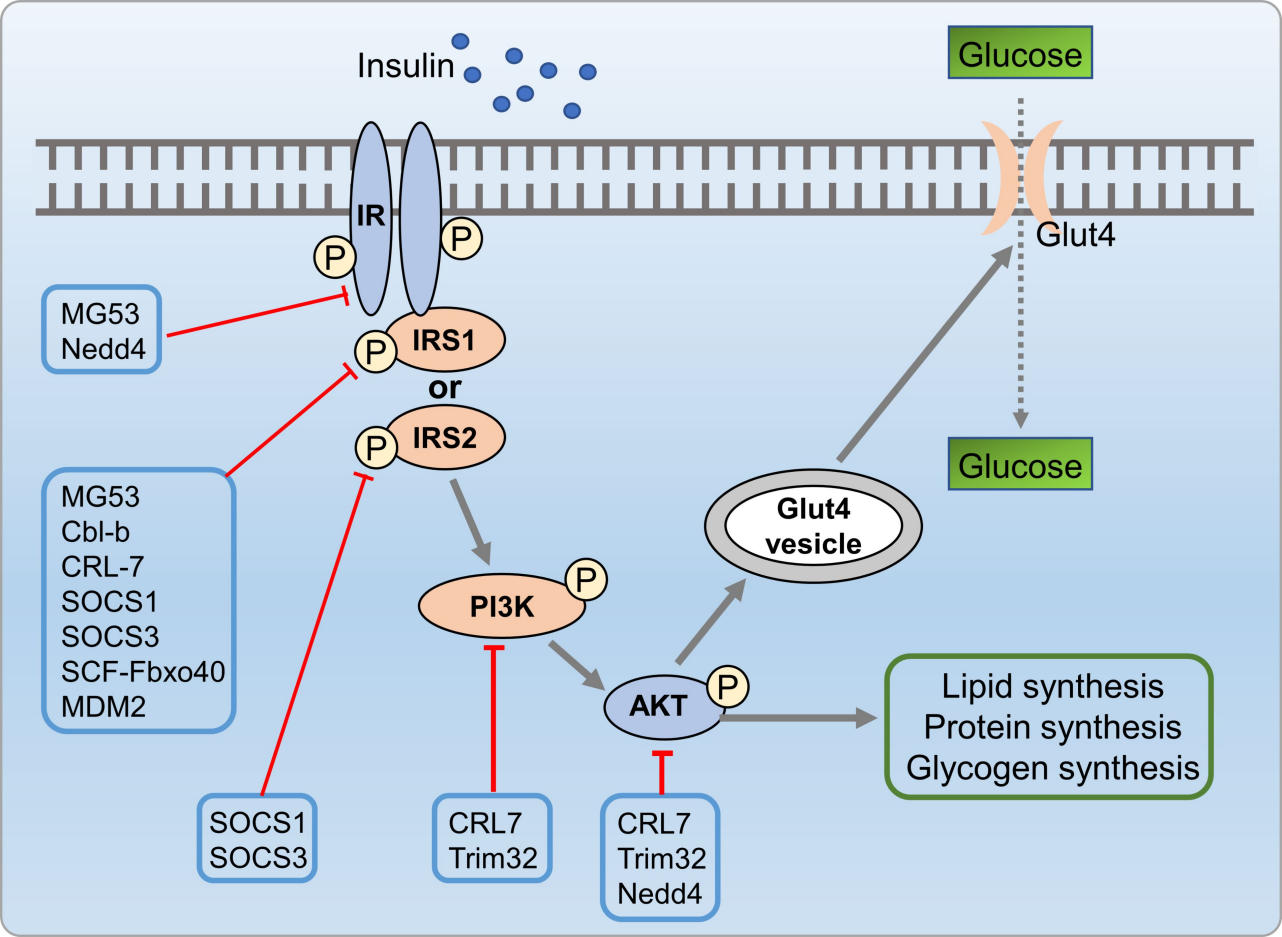
UPS mediated insulin resistance process involved in metabolic disorders. Insulin signaling molecules are directly targeted by E3 ubiquitin ligases such as MG53, CRL7, SOCS 1/3, MDM2, NEDD4, and TRIM32, which are involved in the ubiquitin-dependent degradation of key insulin signaling molecules, including PI3K, AKT, IR, and IRS, leading to insulin resistance.

Insulin resistance is closely associated with development of metabolic disorders (150). Research indicated that UPS is crucial in insulin resistance by regulating insulin signaling (151). Altered E3 ligase expression targeted insulin signaling molecules. Genetic deletion of these ligases can enhance or impair insulin action. Palmitate-induced insulin resistance involves upregulation of E3 ligases, which targeting AKT, IR, and IRS1, leading to ubiquitin-dependent degradation (152). Feeding Ube4A knockout mice with a high-fat diet (HFD) led to an augmented depot of white and brown adipose tissues, exacerbate insulin resistance and inflammation. Ube4A deficiency intensified hepatic steatosis, inflammation, and liver injury in mice. This effect was attributed to K63-linked ubiquitination (K63-Ub) of Akt and APPL1, further promoted insulin-induced AKT activation (153). E3 ubiquitin ligases contribute to insulin resistance via two main mechanisms, including direct targeting of insulin signaling molecules and indirect regulation of insulin signaling by targeting pro-inflammatory mediators. Firstly, insulin signaling molecules were directly targeted by E3 ubiquitin ligases, such as mitsugumin 53 (MG53) (154), CRL7 (155), Cbl proteins (156, 157), suppressors of cytokine signaling (SOCS) 1/3 (158), Fbxo40 (159), murine double minute 2 (MDM2) (160), NEDD4 (161, 162), and TRIM32 (163), which are known to be involved in the ubiquitin-dependent degradation of key insulin signaling molecules, including PI3K, AKT, IR, and IRS, contributed to insulin resistance. Drugs that can protect these molecules against UPS-mediated degradation might hold significant therapeutic promise for addressing insulin resistance syndromes and diabetes. Additionally, E3 ubiquitin ligases indirectly influence insulin signaling by targeting pro-inflammatory mediators linked to insulin resistance (Figure 9).

## 4 Conclusion and perspectives

Metabolic disorders are increasing worldwide and negatively impact global economy and health. At present, the therapeutic treatment options for metabolic disorders are limited. The exclusive involvement of the UPS in metabolic disorders establishes it as a burgeoning subject, holding promise for drug discovery and emerging as a vital focus. Compelling evidence from both *in vivo* and *in vitro* settings demonstrates that natural polyphenols exhibit protective effects across various metabolic disorders, including inhibiting intrahepatic lipid accumulation, mitigating inflammatory factors, possessing antioxidative properties, and attenuating fibrotic progression (164, 165). So far, our understanding of the mechanisms of polyphenols preventing metabolic disorders remains to be further investigated in the future.

Despite notable progress, considerable gaps in comprehending UPS in metabolic disorders persist, necessitating urgent attention to address critical inquiries and effectively bridge knowledge disparities. First, it is crucial to thoroughly grasp the molecular mechanism of action of polyphenols and their potential interaction with the UPS. For example, in future research, we need to conduct more comprehensive and systematic studies to determine whether different polyphenols directly with UPS or indirectly promote the degradation of related proteins or act as proteasome regulators to directly control intracellular proteasome levels. In addition, by characterizing the upstream regulators or downstream targets of UPS, researchers can develop treatment approaches with improved efficacy and specificity. After gaining a deeper understanding of how polyphenols regulate upstream factors or downstream targets of the UPS, we can develop more effective agonists or inhibitors targeting these sites to achieve more precise regulation of metabolic disorders.

In the next testing phase, additional *in vivo* and *in vitro* experiments are crucial to assess molecule bioavailability, unravel their specific mechanism of action, and explore potential interactions with conventional proteasome inhibitors. Furthermore, clinical trials are needed to determine the efficacy and safety of UPS-targeted therapies for treating metabolic disorders in human patients. Future research should focus on developing new approaches to modulate the UPS pathway, including the use of natural compounds such as polyphenols, alongside the creation of small molecule inhibitors that specifically target enzymes within the UPS pathway. Additionally, further exploration is needed on the role of polyphenols in combination with other drugs to improve metabolic disorders. In summary, there is great potential for developing new therapies targeting metabolic disorders based on the regulation of the UPS pathway by polyphenols.

## Author contributions

WG: Data curation, Formal analysis, Resources, Writing – original draft, Writing – review & editing. GW: Data curation, Methodology, Writing – original draft, Writing – review & editing. GC: Conceptualization, Formal analysis, Methodology, Writing – original draft, Writing – review & editing. XM: Data curation, Writing – original draft, Writing – review & editing. ZX: Conceptualization, Funding acquisition, Supervision, Writing – original draft, Writing – review & editing. SC: Conceptualization, Methodology, Project administration, Supervision, Visualization, Writing – original draft, Writing – review & editing.
